# Systematic Elucidation of the Mechanism of *Sappan Lignum* in the Treatment of Diabetic Peripheral Neuropathy Based on Network Pharmacology

**DOI:** 10.1155/2021/5528018

**Published:** 2021-06-29

**Authors:** Xiaomin Kang, De Jin, Yuqing Zhang, Rongrong Zhou, Yuehong Zhang, Fengmei Lian

**Affiliations:** ^1^Guang'anmen Hospital, China Academy of Chinese Medical Sciences, Beijing 100053, China; ^2^Beijing University of Chinese Medicine, Beijing 100029, China

## Abstract

**Background:**

Diabetic peripheral neuropathy (DPN) is one of the most common chronic complications of diabetes, which seriously affects the physical and mental health of patients. *Sappan Lignum* (SL) is effective in treating DPN. Previous reports have shown that SL has a clear hypoglycemic and anti-inflammatory effect. However, the study of SL in the treatment of DPN is still limited and rare.

**Objective:**

To investigate the mechanism of SL in the treatment of DPN based on network pharmacology.

**Methods:**

The active ingredients of SL were screened by related databases. The compound targets were collected by the target prediction platforms. The DPN-related targets were gathered through disease databases. The intersection targets were obtained by uploading the compound targets and disease targets to Venny 2.1.0, and a compound-target network was constructed by Cytoscape3.7.2. The protein-protein interaction (PPI) relationships were obtained by the STRING11.0 database. Genome Ontology (GO) and Kyoto Encyclopedia of Genes and Genomes (KEGG) analyses were performed using the DAVID6.8 database. Molecular docking of key compounds and core targets was performed by DockThor.

**Results:**

A total of 29 compounds and 51 intersection targets with potential therapeutic effects on DPN were obtained. The compound-target network construction resulted in four key compounds: protostemonine, 3-deoxysappanchalcone, 7,3′,4′-trihydroxy-3-benzyl-2H-chromene, and o-12′-methylergocornine. PPI network analysis yielded 10 core targets: AKT1, MAPK3, CXCL8, TNF, OPRM1, MTOR, STAT3, MAPK8, SIRT1, and HSP90AA1. KEGG analysis resulted in 82 signaling pathways (*P* < 0.05), including insulin resistance, HIF-1 signaling pathway, and type II diabetes. The docking results indicated that the main active compounds could stably bind to core targets.

**Conclusion:**

SL had the mechanism of multiple ingredients, multiple targets, and multiple pathways in the treatment of DPN. This study provided a scientific basis for further research on the treatment of DPN with SL and its extracts.

## 1. Introduction

DPN is one of the most common chronic complications of diabetes mellitus. The main clinical manifestations of DPN are limb pain, numbness, and paresthesia. Severe cases may be associated with peripheral vascular disease and infection-causing foot ulcers or gangrene, resulting in amputation or disability, threatening the quality of life and physical and mental health of patients. Because the pathogenesis has not been fully elucidated, the current clinical treatment of DPN is mainly to relieve clinical symptoms, including control of blood glucose, repair of nerves, antioxidative stress, improvement of microcirculation, and improvement of metabolic disorders [1].

*Sappan Lignum* (SL) is the dried heartwood of *Caesalpinia sappan* L. of Leguminosae, which has the effects of activating blood circulation, removing blood stasis, relieving swelling, and relieving pain. Modern studies have shown that SL has pharmacological effects such as anti-inflammatory, antitumor, antiviral, antimicrobial, and antioxidant effects [2].

SL has definite efficacy in the treatment of DPN, and Chinese herbal compound decoctions containing SL for the treatment of DPN have been widely used in clinical practice. Previous reports have shown that SL had a clear hypoglycemic and anti-inflammatory effect [3–5]. However, studies on SL in the treatment of DPN are still limited and few.

Network pharmacology [6] is a branch of pharmacology that uses network methods to analyze the “multicomponent, multitarget, and multipathway” synergistic relationship between drugs and diseases and targets. Through the interdisciplinary application and the analysis and reintegration of the overall biological network, the relationship between drugs and targets of action can be revealed, the synergistic mechanism between the active ingredients of traditional Chinese medicine can be explained, and the microbiological basis of the efficacy of traditional Chinese medicine can be confirmed [7].

Therefore, this study, based on network pharmacology, aims to investigate the mechanism of SL in the treatment of DPN. It provides a scientific basis for further research on the treatment of DPN with SL or its extracts and also provides a reference for the secondary development of SL.

**Figure 1:**
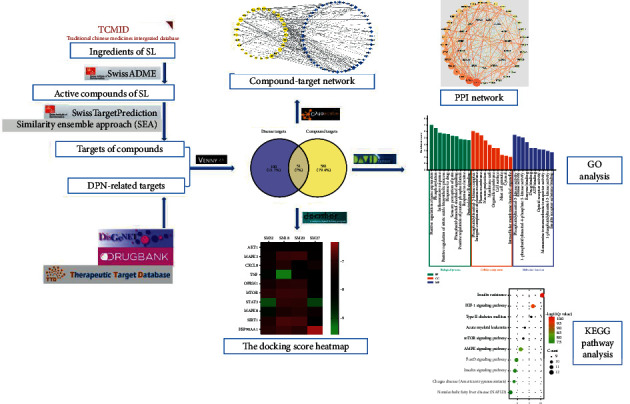
The workflow of our study.

In this paper, based on network pharmacology, the mechanism of SL in the treatment of DPN was explored. Firstly, the ingredients of SL were searched through TCMID, and its active ingredients were screened by SwissADME. The active ingredients were imported into SwissTargetPrediction or SEA library to predict their targets. TTD, DrugBank, and DisGeNET databases were used to search for relevant targets of DPN. The intersection targets were obtained by uploading the ingredient targets and disease targets to Venny 2.1.0, and a compound-target (gene) network was constructed by Cytoscape3.7.2. The intersection targets were imported into the STRING11.0 database to build a PPI network. GO functional enrichment analysis and KEGG pathway enrichment analysis of intersection targets were acquired from the DAVID6.8 database. Molecular docking validation of key compounds and core targets was performed by DockThor. The workflow is summarized in Figure 1.

## 2. Materials and Methods

### 2.1. Acquisition of Active Ingredients and Prediction of Target

The ingredients of SL were obtained from TCMID (https://www.megabionet.org/tcmid/) through using “SUMU” as the search term. The SMILE numbers of the ingredients were imported into SwissADME [8] (http://www.swissadme.ch/) platform, which selects ingredients with high gastrointestinal absorption and drug-likeness column containing two or more “Yes” as the active ingredients of SL. The SMILE numbers of the active ingredients were uploaded to SwissTargetPrediction [9] (http://www.swisstargetprediction.ch/) to predict the relevant target. SEA library [10] (http://sea.bkslab.org/) was used as a supplement.

### 2.2. Collection of DPN-Related Targets

In the TTD [11] (http://db.idrblab.net/ttd/), DrugBank (https://www.drugbank.ca/), and DisGeNET (https://www.disgenet.org/) databases, “diabetic neuropathy,” “diabetic peripheral neuropathy,” and so forth were searched as keywords to obtain targets related to DPN, and the collected targets were combined and duplicates removed.

### 2.3. Acquisition of Intersection Target

The ingredient targets of SL and the DPN-related targets were uploaded to Venny 2.1.0 (https://bioinfogp.cnb.csic.es/tools/venny/) for intersection to obtain the potential targets of SL for the treatment of DPN.

### 2.4. Construction and Analysis of Compound-Target (Gene) Network

The information of the active compounds of SL and the corresponding targets of action was imported into Cytoscape3.7.2 software to construct a compound-target (gene) network. The topological properties of the network were analyzed, and the results were visualized.

### 2.5. Construction and Analysis of PPI

The intersection targets were uploaded to the STRING11.0 (https://string-db.org/) database to obtain protein interaction relationships, and the results were imported into Cytoscape3.7.2 software to establish a PPI network and obtain visual analysis results.

### 2.6. GO and KEGG Analysis

DAVID6.8 (https://david.ncifcrf.gov/) database is an online tool for GO enrichment analysis and KEGG pathway annotation analysis of the target. The intersection targets were imported into the DAVID6.8 database for GO enrichment analysis and KEGG pathway enrichment analysis, and the potential signaling pathways and mechanisms of action of SL in the treatment of DPN were screened using a threshold (*P* < 0.05).

### 2.7. Molecular Docking Verification

The screened key compounds were subjected to molecular docking with core target proteins to verify the efficacy of the compounds. PDB format files of core target proteins were downloaded from the Protein Data Bank (PDB) (http://www.rcsb.org/pdb/home/home.do). SDF format files of the three-dimensional structures of the compounds were downloaded from the PubChem (https://pubchem.ncbi.nlm.nih.gov/) platform. Finally, compounds and proteins were uploaded to DockThor [12] (https://dockthor.lncc.br/v2/) to perform online molecular docking; then the docking results were obtained and visualized.

## 3. Results

### 3.1. Active Ingredients and Related Targets

A total of 62 ingredients were obtained from TCMID by searching “SUMU.” The components without SMILE numbers whose targets could not be obtained after consulting the literature were removed. The components with low gastrointestinal absorption and poor drug-likeness were excluded. The compounds with no target identified by SMILE number in SwissTargetPrediction or SEA library were deleted. For the compounds with the same SMILE number, only one of them was retained. Finally, a total of 39 compounds and 632 corresponding targets were obtained. For the convenience of subsequent studies, the active compounds of SL were numbered SM1-SM39, respectively, as detailed in Table 1.

### 3.2. DPN-Related Targets

A total of 151 DPN-related targets were obtained from TTD, DrugBank, and DisGeNET, after removing the targets without UniProt ID or duplicated targets.

### 3.3. Intersection Targets of Disease Targets and Compound Targets

**Figure 2:**
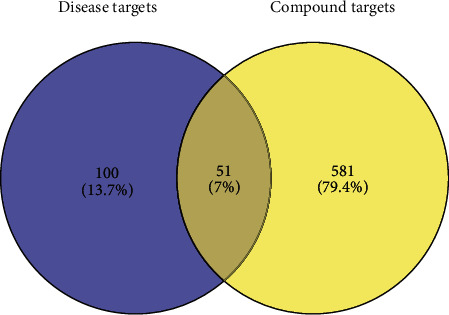
The Venn diagram of disease targets and compound targets.

The compound targets and DPN-related targets were imported into Venny 2.1.0 to take the intersection. 51 intersection targets were obtained and a Venny diagram was generated. As Figure 2 shows, yellow represents the compound targets and purple represents the DPN-related targets. The 51 intersection targets are supplied in Table S1.

### 3.4. Compound-Target (Gene) Network

**Figure 3:**
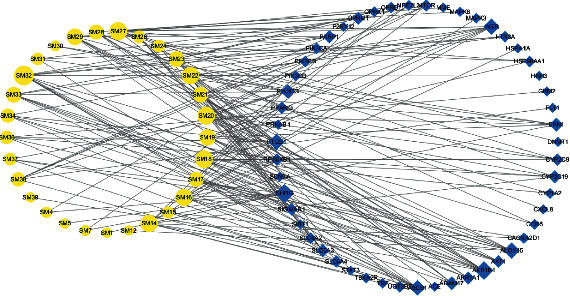
The compound-target (gene) network.

The information of SL components and corresponding targets was imported into Cytoscape3.7.2 software to construct a compound-target network and obtain a visual analysis result, as detailed in Figure 3. The network contains 80 nodes and 178 edges. Yellow circular nodes represent the SL active components, and blue diamond nodes represent the targets of action. The higher the Degree value, the more important it is in the network. From Figure 3, it can be seen that the same component can correspond to multiple targets, and the same target can correspond to multiple components, intuitively reflecting the action characteristics of multiple components and multiple targets of SL. In addition, 29 active components of SL have potential therapeutic effects on DPN. Among them, the components protostemonine (SM32), 3-deoxysappanchalcone (SM18), 7,3′,4′-trihydroxy-3-benzyl-2H-chromene (SM20), and o-12′-methylergocornine (SM27) correspond to the most potential targets, suggesting that SM32, SM18, SM20, and SM27 may be potential active ingredients of SL in the treatment of DPN.

### 3.5. PPI Network of Intersection Targets

**Figure 4:**
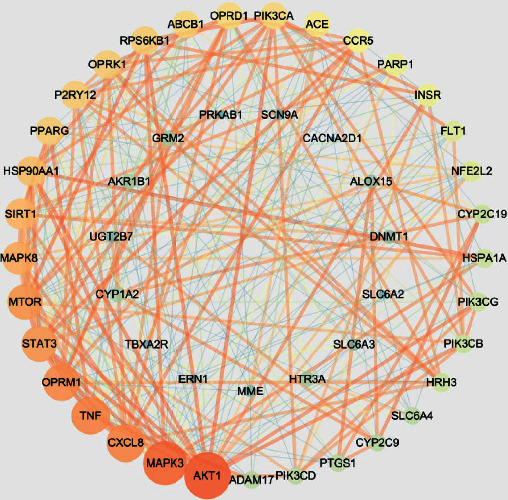
The PPI network of intersection targets.

Protein targets were uploaded to the STRING11.0 database to obtain protein-protein interaction relationships, and the results were imported into Cytoscape3.7.2 software for visual analysis to construct a PPI network. The results of the topological analysis showed that the mean values of BetweennessCentrality, ClosenessCentrality, and Degree were 0.03, 0.53, and 11.2, respectively. As detailed in Figure 4, a total of 48 nodes with 268 edges (SHBG, SIGMAR1, and AKR1A1 were not embodied in the interaction network because they did not interact with other proteins) were obtained. The size and color of the node reflect the Degree value of the node. The change of node from large to small and the change of node from red to green correspond to the change of Degree value from high to low. The thickness and color of the edges reflect the Combine score. The change of edge from thick to fine and the change of edge from red to green correspond to the change of Combine score from high to low. The larger the node and the thicker the edge, the closer the interaction relationship between target proteins. According to the ranking of Degree values of nodes, the top ten were AKT1, MAPK3, CXCL8, TNF, OPRM1, MTOR, STAT3, MAPK8, SIRT1, and HSP90AA1. These are hub nodes of the network and may be core targets of SL for the treatment of DPN.

### 3.6. GO and KEGG Analysis

The intersection targets were imported into the DAVID6.8 database for GO enrichment analysis and KEGG pathway enrichment analysis, and the results were screened using a threshold (*P* < 0.05).

**Figure 5:**
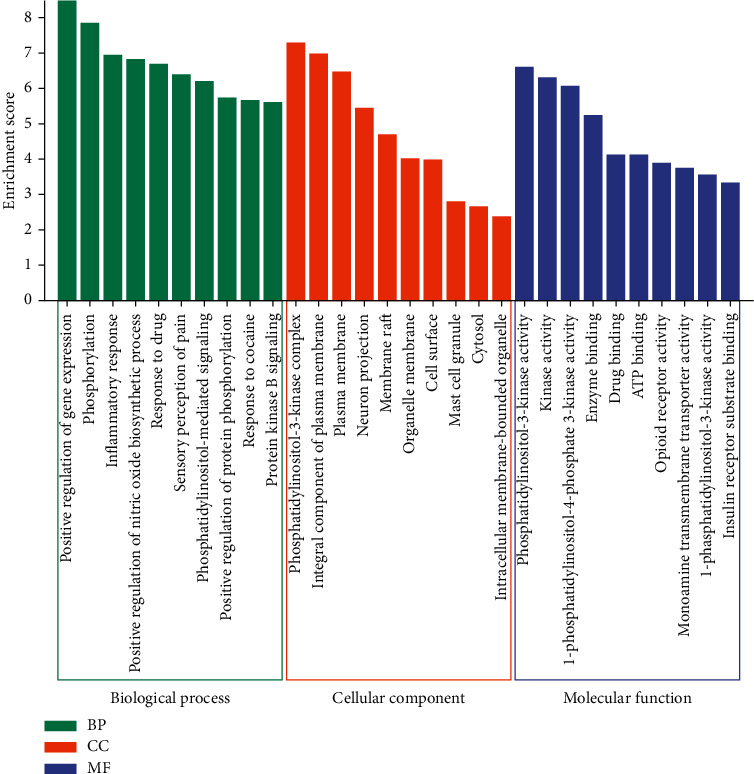
The top 10 GO terms of BP, CC, and MF, respectively.

GO functional enrichment analysis yielded 216 GO terms (*P* < 0.05), including 155 BP terms, 23 CC items, and 38 MF terms.  Figure 5 showed the top 10 most significantly enriched GO terms, respectively. In terms of BP, these targets were mainly concerned with positive regulation of gene expression, phosphorylation, inflammatory response, positive regulation of nitric oxide biosynthetic process, response to the drug, sensory perception of pain, phosphatidylinositol-mediated signaling, positive regulation of protein phosphorylation, response to cocaine, and protein kinase B signaling. In terms of CC, the targets were mainly related to the cell membrane, plasma membrane, cytoplasm, and organelles. In terms of MF, the targets were mainly related to phosphatidylinositol-kinase activity, enzyme binding, drug binding, ATP binding, opioid receptor activity, monoamine transmembrane transporter activity, and insulin receptor substrate binding.

**Figure 6:**
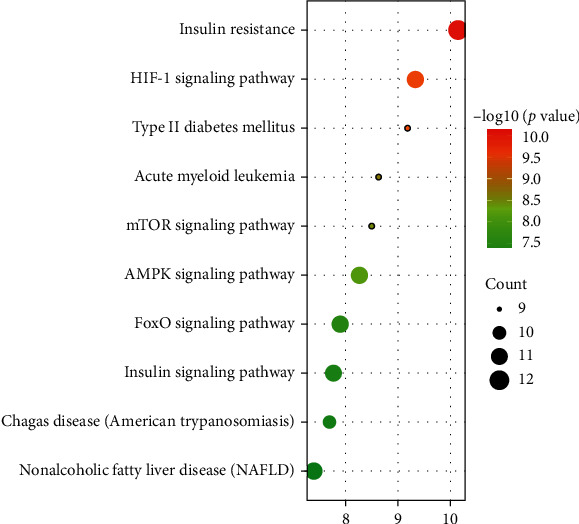
The top 10 signaling pathways of KEGG enrichment analysis.

KEGG pathway enrichment analysis yielded 82 signaling pathways (*P* < 0.05). As shown in Figure 6, a bubble diagram was drawn by listing the top 10 signal pathways according to the *P* value. The top 10 signaling pathways were insulin resistance, HIF-1 signaling pathway, type II diabetes mellitus, acute myeloid leukemia, mTOR signaling pathway, AMPK signaling pathway, FoxO signaling pathway, insulin signaling pathway, Chagas disease (American trypanosomiasis), and nonalcoholic fatty liver disease (NAFLD).

### 3.7. Molecular Docking

**Figure 7:**
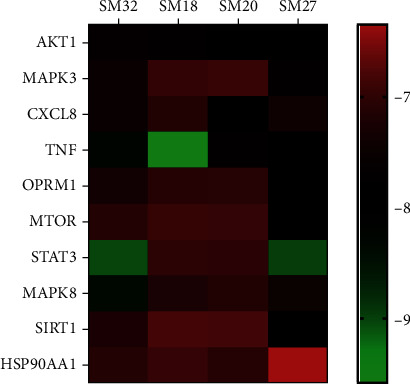
The docking scores between ligands and receptors.

Molecular docking validation was performed by DockThor between the top 4 compounds in the compound-target network according to Degree value and the top 10 core target proteins in the PPI network. The binding degree of protein compound can be judged by the affinity score in the molecular docking results. In general, the lower the affinity score, the more stable the conformation of ligand binding to the receptor. As shown in Figure 7, the binding scores of compounds to core target proteins were lower than −6.343 (kcal/mol), indicating that the main active compounds could stably bind to core targets and play a role in the therapeutic effect of DPN. In addition, the docking scores of TNF and AKT1 with the four compounds were lower than the average, indicating that TNF with ATK1 may be the important targets for the treatment of DPN with SL. The docking scores are supplied in Table S2.

## 4. Discussion

Based on the network pharmacological screening, 29 active compounds of SL had a potential role in the treatment of DPN. According to the analysis of the compound-target network, the top 4 compounds were protostemonine, 3-deoxysappanchalcone, 7,3′,4′-trihydroxy-3-benzyl-2H-chromene, and o-12′-methylergocornine.

Studies have shown that protostemonine (PSN) is an anti-inflammatory alkaloid. PSN significantly inhibited lipopolysaccharide- (LPS-) induced MAPKs and AKT phosphorylation, iNOS expression, and NO production in macrophages. Meanwhile, PSN markedly attenuated LPS-induced inflammatory cell infiltration and reduced the production of proinflammatory cytokines (TNF-*α*, IL-1*β*, and IL-6) [13, 14].

It has been experimentally shown that 3-deoxysappanchalcone (3-DSC) played a role in the anti-inflammatory effect by inducing heme oxygenase-1 (HO-1) expression via activating the AKT/mTOR pathway. 3-DSC inhibited the production of NO and IL-6 by LPS-stimulated RAW264.7 cells; and it was considered as a valuable compound for modulating inflammatory conditions [15]. In addition, it has been shown that 3-DSC potentiated IL-6-induced phosphorylation and subsequent transactivation of signal transducer and activator of transcription-3 (STAT3), thereby increasing the expression of cyclin-dependent kinase-4 (Cdk4), fibroblast growth factor (FGF), and vascular endothelial growth factor (VEGF) [16]. Studies also suggested that 3-DSC attenuated the inflammatory response by suppressing CCL5 and CXCL10 secretions in endothelial cells and reducing the production of IL-6 and IL-1-beta in monocytes/macrophages [17].

7,3′,4′-Trihydroxy-3-benzyl-2H-chromene is a homoisoflavan [18]. Noshita et al.'s study evaluated homoisoflavonoids as AChE inhibitors and neurite growth promoters, and the results showed that some of the homoisoflavonoids significantly promoted neurite outgrowth induced by nerve growth factor (NGF) [19]. Studies have shown that homoisoflavan had a good *α*-glucosidase and COX-II inhibitory activity [20]. Studies suggested that homoisoflavans exhibited moderate inhibition of NO production in LPS-stimulated BV-2 microglial cells [21].

O-12′-methylergocornine is an ergot alkaloid. It has been shown that ergot alkaloids are vasoactive and can potentially alter arterial blood supply and venous drainage from the bovine foregut [22]. It has also been suggested that ergocornine is a 5-HT receptor stimulant, and the new ergoline derivative may represent a new class of antidepressant drugs that act by releasing extragranular 5-HT stores [23].

The PPI network illustrated that AKT1, MAPK3, CXCL8, TNF, OPRM1, MTOR, STAT3, MAPK8, SIRT1, and HSP90AA1 were the top 10 protein targets; and these targets may be the main targets of SL in the treatment of DPN.

AKT1 was one of 3 closely related serine/threonine-protein kinases (AKT1, AKT2, and AKT3) called the AKT kinase. AKT was responsible for the regulation of glucose uptake by mediating insulin-induced translocation of the SLC2A4/GLUT4 glucose transporter to the cell surface [24]. AKT played a role as a key modulator of the AKT-mTOR signaling pathway controlling the tempo of the process of newborn neuron integration during adult neurogenesis, including correct neuron positioning, dendritic development, and synapse formation. AKT mediated the effects of various growth factors such as platelet-derived growth factor (PDGF), epidermal growth factor (EGF), insulin, and insulin-like growth factor I (IGF-I) [25, 26]. MAPK3 mediated diverse biological functions such as cell growth, adhesion, survival, and differentiation through the regulation of transcription, translation, and cytoskeletal rearrangements [27, 28]. CXCL8 was a proinflammatory cytokine that promotes neutrophil migration [29]. Li et al.'s study suggested that CXCL8 functioned as an important autocrine growth and angiogenic factor in regulating multiple biological activities in endothelial cells [30]. TNF induced insulin resistance in adipocytes by inhibiting insulin-induced IRS1 tyrosine phosphorylation and insulin-induced glucose uptake and played a role in angiogenesis by inducing VEGF production synergistically with IL-1B and IL-6 [31]. The reduced function of TNF and its major proinflammatory receptor TNFR1 was a risk factor for the development of multiple sclerosis [32]. The mu-opioid receptor (MOR), encoded by OPRM1, regulated the analgesic response to pain [33]. mTOR was a central regulator of cellular metabolism, growth, and survival in response to growth factors, nutrients, energy, and stress signals [34]. mTOR has been repeatedly shown to participate in neuronal development and the proper functioning of mature neurons [35]. It has been shown that STAT3 regulating islet cell VEGF-A was required for the normal development and maintenance of the endocrine pancreas and islet microvascular network [36]. STAT3 had a critical role in regulating cell fate, inflammation, and immunity [37]. MAPK8 signaling to STMN family proteins has been implicated specifically in neuronal maturation, degeneration, and cell stress responses [38]. SIRT1 deacetylated FOXO3 in response to oxidative stress, thereby increasing its ability to induce cell cycle arrest and resistance to oxidative stress but inhibiting the induction of FOXO3-mediated apoptotic transcriptional activity [39]. HSP90AA1 was one of the main mediators of activation by bacterial lipopolysaccharide [40]. In summary, the main targets were significant in DPN. A target-interaction network for the action of SL in the treatment of DPN was constructed, which provided a scientific basis for elaborating the mechanism of SL in the treatment of DPN via multiple targets and multiple pathways.

DPN is an endocrine system disease with neuropathy and microangiopathy, involving multiple metabolic pathways. The top 10 DPN-related metabolic pathways, obtained by KEGG enrichment analysis of intersection targets, included insulin resistance, type II diabetes, and insulin signaling pathways, indicating that one of the main mechanisms of SL in the treatment of DPN is the regulation of blood glucose. For HIF-1 signaling pathway, hypoxia-inducible factor 1 (HIF-1) is an oxygen-regulated transcriptional activator involved in the regulation of oxygen in the cellular environment. Oxidative stress often causes neuronal dysfunction and cell death. Activation of the HIF-1 signaling pathway is one of the key mechanisms of cellular defense against oxidative stress [41]. For mTOR signaling pathway, previous reports indicated that mTOR signaling pathway affected multiple metabolic parameters, including cellular metabolic homeostasis, insulin resistance, insulin secretion, stem cell proliferation and differentiation, and pancreatic *β*-cell function [42]. It has been shown that mTOR signaling could mediate local protein synthesis that was critical for the growth of axons and dendrites and played a key role in diabetic small fiber neuropathy [43]. For AMPK signaling pathway, studies have demonstrated that activation of the AMPK signaling pathway could significantly increase motor nerve conduction velocity and decrease the level of inflammatory cytokines. The activation of the AMPK signaling pathway in diabetic neuropathy might be associated with the anti-inflammatory response [44]. For FoxO signaling pathway, it has been shown that FoxO signaling pathways can regulate insulin signaling, gluconeogenesis, insulin resistance, immune cell migration, and cellular senescence and can also control cell fate through oxidative stress, autophagy, and apoptotic pathways [45].

In summary, this study clarified the action mode of multiple compounds, multiple targets, and multiple pathways of SL in the treatment of DPN. A total of 29 active ingredients and 51 relevant targets of SL for the treatment of DPN were selected. The effectiveness of the binding of the top 4 key compounds to the top 10 core target proteins was verified by molecular docking. According to the results of the PPI network and KEGG analysis, it is considered that SL is mainly used to treat DPN by regulating blood glucose, resisting inflammation, resisting oxidative stress, nourishing nerves, and repairing vascular injury.

## 5. Conclusion

Based on the action mode of multiple compounds, multiple targets, and multiple pathways of traditional Chinese medicine, following the principles of network pharmacology, we systematically explored the mechanism of action of SL in the treatment of DPN. This study provided a scientific basis for further research on the treatment of DPN with SL or its active ingredients, as well as a reference for the secondary development of SL.

## Figures and Tables

**Table 1:** Active compounds of SL.

No.	Compound name
SM1	10-o-Methylprotosappanin B
SM2	3′-Deoxy-4-o-methylsappanol
SM3	3′-Deoxysappanol
SM4	3′-o-Methylsappanol
SM5	3′-o-Methylbrazilin
SM6	3,7-Dihydroxychroman-4-one
SM7	4-o-Methylsappanol
SM8	Brazilin
SM9	Hematein
SM10	Protosappanin B
SM11	Protosappanin dimethyl acetal
SM12	Sappanol
SM13	Sarcosine
SM14	(-)-Methyl selina-3,11-dien-14-oate
SM15	(e)-2-Nonenal
SM16	2′-Methoxy-3,4,4′-trihydroxychalcone
SM17	3′-Methoxy-4′,5,7-trihydroxyflavone
SM18	3-Deoxysappanchalcone
SM19	3-Deoxysappanone B
SM20	7,3′,4′-Trihydroxy-3-benzyl-2H-chromene
SM21	7-Hydroxy-3-(4′-hydroxybenzylidene)-chroman-4-one
SM22	Bonducellin
SM23	d-n-Methyl-pseudoephedrine
SM24	Dibenzoxocin
SM25	Gallic acid
SM26	Menthol
SM27	o-12′-Methyl ergocornine
SM28	Oleic acid
SM29	Ombuin
SM30	Protosappanin A
SM31	Protosappanin A dimethyl acetal
SM32	Protostemonine
SM33	Quercetin
SM34	Sappanchalcone
SM35	Sappanin
SM36	Sappanone B
SM37	Stearic acid
SM38	Tetraacetyl brazilin
SM39	Methyl brevifolincarboxylate

## Data Availability

The data used to support the findings of this study are available from the corresponding author upon request.

## Supplementary Materials

Supplementary Materials Table S1: the information of 51 intersection targets. Table S2: molecular docking scores (kcal/mol).

## References

[1] Jia W., Weng J., Zhu D. (2019). Standards of medical care for type 2 diabetes in China 2019. *Diabetes/Metabolism Research and Reviews*.

[2] Jing W., Zhang X., Zhou H. (2019). Naturally occurring cassane diterpenoids (CAs) of Caesalpinia: a systematic review of its biosynthesis, chemistry and pharmacology. *Fitoterapia*.

[3] Wediasari F., Nugroho G. A., Fadhilah Z., Elya B., Setiawan H., Mozef T. (2020). Hypoglycemic effect of a combined *Andrographis paniculata* and *Caesalpinia sappan* extract in streptozocin-induced diabetic rats. *Advances in Pharmacological and Pharmaceutical Sciences*.

[4] Chakrabarti S., Biswas T. K., Rokeya B. (2003). Advanced studies on the hypoglycemic effect of *Caesalpinia bonducella* F. in type 1 and 2 diabetes in Long Evans rats. *Journal of Ethnopharmacology*.

[5] Rizk M. Z., Aly H. F., Abo-Elmatty D. M., Desoky M., Ibrahim N., Younis E. A. (2016). Hepatoprotective effect of *Caesalpinia gilliesii* and *Cajanus cajan* proteins against acetoaminophen overdose-induced hepatic damage. *Toxicology and Industrial Health*.

[6] Hopkins A. L. (2008). Network pharmacology: the next paradigm in drug discovery. *Nature Chemical Biology*.

[7] Luo T. T., Lu Y., Yan S.-K., Xiao X., Rong X.-L., Guo J. (2020). Network pharmacology in research of Chinese medicine formula: methodology, application and prospective. *Chinese Journal of Integrative Medicine*.

[8] Daina A., Michielin O., Zoete V. (2017). SwissADME: a free web tool to evaluate pharmacokinetics, drug-likeness and medicinal chemistry friendliness of small molecules. *Scientific Reports*.

[9] Daina A., Michielin O., Zoete V. (2019). SwissTargetPrediction: updated data and new features for efficient prediction of protein targets of small molecules. *Nucleic Acids Research*.

[10] Keiser M. J., Roth B. L., Armbruster B. N., Ernsberger P., Irwin J. J., Shoichet B. K. (2007). Relating protein pharmacology by ligand chemistry. *Nature Biotechnology*.

[11] Wang Y., Zhang S., Li F. (2020). Therapeutic target database 2020: enriched resource for facilitating research and early development of targeted therapeutics. *Nucleic Acids Research*.

[12] Santos K. B., Guedes I. A., Karl A. L. M., Dardenne L. E. (2020). Highly flexible ligand docking: benchmarking of the DockThor program on the LEADS-PEP protein-peptide data set. *Journal of Chemical Information and Modeling*.

[13] Wu Y., Nie Y., Huang J. (2019). Protostemonine alleviates heat-killed methicillin-resistant *Staphylococcus aureus*-induced acute lung injury through MAPK and NF-*κ*B signaling pathways. *International Immunopharmacology*.

[14] Wu Y.-X., He H.-Q., Nie Y.-J., Ding Y.-H., Sun L., Qian F. (2018). Protostemonine effectively attenuates lipopolysaccharide-induced acute lung injury in mice. *Acta Pharmacologica Sinica*.

[15] Kim J.-H., Choo Y.-Y., Tae N., Min B.-S., Lee J.-H. (2014). The anti-inflammatory effect of 3-deoxysappanchalcone is mediated by inducing heme oxygenase-1 via activating the AKT/mTOR pathway in murine macrophages. *International Immunopharmacology*.

[16] Kim Y. E., Choi H. C., Lee I.-C., Yuk D. Y., Lee H., Choi B. Y. (2016). 3-Deoxysappanchalcone promotes proliferation of human hair follicle dermal papilla cells and hair growth in C57BL/6 mice by modulating WNT/*β*-Catenin and STAT signaling. *Biomolecules & Therapeutics*.

[17] Yang F., Zhou W.-L., Liu A.-L. (2012). The protective effect of 3-deoxysappanchalcone on in vitro influenza virus-induced apoptosis and inflammation. *Planta Medica*.

[18] Zhao H., Bai H., Wang Y., Li W., Koike K. (2008). A new homoisoflavan from *Caesalpinia sappan*. *Journal of Natural Medicines*.

[19] Noshita T., Fujita K., Koga T., Ouchi H., Tai A. (2021). Synthesis and biological activity of (±)-7,3′,4′-trihydroxyhomoisoflavan and its analogs. *Bioorganic & Medicinal Chemistry Letters*.

[20] Helal I. E., Elsbaey M., Zaghloul A. M., Mansour E.-S. S. (2021). A new homoisoflavan from *Dracaena cinnabari* Balf. f. resin: *α*-glucosidase and COX-II inhibitory activity. *Natural Product Research*.

[21] Pang D.-R., Pan B., Sun J. (2018). Homoisoflavonoid derivatives from the red resin of *Dracaena cochinchinensis*. *Fitoterapia*.

[22] Foote A. P., Harmon D. L., Strickland J. R., Bush L. P., Klotz J. L. (2011). Effect of ergot alkaloids on contractility of bovine right ruminal artery and vein 1, 2. *Journal of Animal Science*.

[23] Corrodi H., Farnebo L.-O., Fuxe K., Hamberger B. (1975). Effect of ergot drugs on central 5-hydroxytryptamine neurons: evidence for 5-hydroxytryptamine release or 5-hydroxytryptamine receptor stimulation. *European Journal of Pharmacology*.

[24] Kane S., Sano H., Liu S. C. H. (2002). A method to identify serine kinase substrates. *Journal of Biological Chemistry*.

[25] Thomas C. C., Deak M., Alessi D. R., Van Aalten D. M. F. (2002). High-resolution structure of the pleckstrin homology domain of protein kinase b/Akt bound to phosphatidylinositol (3,4,5)-trisphosphate. *Current Biology*.

[26] Milburn C. C., Deak M., Kelly S. M., Price N. C., Alessi D. R., Van Aalten D. M. F. (2003). Binding of phosphatidylinositol 3,4,5-trisphosphate to the pleckstrin homology domain of protein kinase B induces a conformational change. *Biochemical Journal*.

[27] Rajalingam K., Wunder C., Brinkmann V. (2005). Prohibitin is required for Ras-induced Raf-MEK-ERK activation and epithelial cell migration. *Nature Cell Biology*.

[28] Chen H., Bai J., Ye J. (2007). JWA as a functional molecule to regulate cancer cells migration via MAPK cascades and F-actin cytoskeleton. *Cellular Signalling*.

[29] Dunlevy J. R., Couchman J. R. (1995). Interleukin-8 induces motile behavior and loss of focal adhesions in primary fibroblasts. *Journal of Cell Science*.

[30] Li A., Varney M. L., Valasek J., Godfrey M., Dave B. J., Singh R. K. (2005). Autocrine role of interleukin-8 in induction of endothelial cell proliferation, survival, migration and MMP-2 production and angiogenesis. *Angiogenesis*.

[31] Nakahara H., Song J., Sugimoto M. (2003). Anti-interleukin-6 receptor antibody therapy reduces vascular endothelial growth factor production in rheumatoid arthritis. *Arthritis & Rheumatism*.

[32] Probert L. (2015). TNF and its receptors in the CNS: the essential, the desirable and the deleterious effects. *Neuroscience*.

[33] Crist R. C., Berrettini W. H. (2014). Pharmacogenetics of OPRM1. *Pharmacology Biochemistry and Behavior*.

[34] Xu L., Brink M. (2016). mTOR, cardiomyocytes and inflammation in cardiac hypertrophy. *Biochimica et Biophysica Acta (BBA)—Molecular Cell Research*.

[35] Switon K., Kotulska K., Janusz-Kaminska A., Zmorzynska J., Jaworski J. (2017). Molecular neurobiology of mTOR. *Neuroscience*.

[36] Kostromina E., Gustavsson N., Wang X. (2010). Glucose intolerance and impaired insulin secretion in pancreas-specific signal transducer and activator of transcription-3 knockout mice are associated with microvascular alterations in the pancreas. *Endocrinology*.

[37] Niu J., Sun Y., Chen B. (2019). Fatty acids and cancer-amplified ZDHHC19 promote STAT3 activation through S-palmitoylation. *Nature*.

[38] Yip Y. Y., Yeap Y. Y. C., Bogoyevitch M. A., Ng D. C. H. (2014). Differences in c-Jun N-terminal kinase recognition and phosphorylation of closely related stathmin-family members. *Biochemical and Biophysical Research Communications*.

[39] Brunet A., Sweeney L. B., Sturgill J. F. (2004). Stress-dependent regulation of FOXO transcription factors by the SIRT1 deacetylase. *Science*.

[40] Triantafilou K., Triantafilou M., Dedrick R. L. (2001). A CD14-independent LPS receptor cluster. *Nature Immunology*.

[41] Lin-Holderer J., Li L., Gruneberg D., Marti H. H., Kunze R. (2016). Fumaric acid esters promote neuronal survival upon ischemic stress through activation of the Nrf2 but not HIF-1 signaling pathway. *Neuropharmacology*.

[42] Maiese K. (2016). Novel nervous and multi-system regenerative therapeutic strategies for diabetes mellitus with mTOR. *Neural Regeneration Research*.

[43] Wu L.-Y., Li M., Qu M.-L. (2018). High glucose up-regulates semaphorin 3A expression via the mTOR signaling pathway in keratinocytes: a potential mechanism and therapeutic target for diabetic small fiber neuropathy. *Molecular and Cellular Endocrinology*.

[44] Hasanvand A., Amini-khoei H., Hadian M.-R. (2016). Anti-inflammatory effect of AMPK signaling pathway in rat model of diabetic neuropathy. *Inflammopharmacology*.

[45] Maiese K. (2015). FoxO transcription factors and regenerative pathways in diabetes mellitus. *Current Neurovascular Research*.

