# Supplementary material for: Systematic Elucidation of the Mechanism of Sappan Lignum in the Treatment of Diabetic Peripheral Neuropathy Based on Network Pharmacology
Source: Evid Based Complement Alternat Med. 2021 Jun 29;2021:5528018. doi: 10.1155/2021/5528018 (PMC8263209; doi:10.1155/2021/5528018)
Supplement: Supplementary Materials — Table S1: the information of 51 intersection targets. Table S2: molecular docking scores (kcal/mol). [file 5528018.f1.zip › 5528018.f1/Table S1.docx]

**Table S1 The information of 51** **intersection targets**

| **Target symbol** | **Uniprot ID** | **Target symbol** | **Uniprot ID** |
| --- | --- | --- | --- |
| ACE | P12821 | CCR5 | P51681 |
| TNF | P01375 | PARP1 | P09874 |
| NFE2L2 | Q16236 | CXCL8 | P10145 |
| PIK3CB | P42338 | INSR | P06213 |
| PIK3CD | O00329 | MME | P08473 |
| PIK3CG | P48736 | GRM2 | Q14416 |
| PPARG | P37231 | HSP90AA1 | P07900 |
| PIK3CA | P42336 | AKR1B1 | P15121 |
| DNMT1 | P26358 | HSPA1A | P0DMV8 |
| PRKAB1 | Q9Y478 | PTGS1 | P23219 |
| MAPK3 | P27361 | HRH3 | Q9Y5N1 |
| MAPK8 | P45983 | TBXA2R | P21731 |
| STAT3 | P40763 | SLC6A2 | P23975 |
| ADAM17 | P78536 | SLC6A3 | Q01959 |
| P2RY12 | Q9H244 | CYP1A2 | P05177 |
| RPS6KB1 | P23443 | ABCB1 | P08183 |
| SCN9A | Q15858 | OPRM1 | P35372 |
| SHBG | P04278 | OPRK1 | P41145 |
| AKT1 | P31749 | OPRD1 | P41143 |
| ERN1 | O75460 | HTR3A | P46098 |
| FLT1 | P17948 | UGT2B7 | P16662 |
| SIRT1 | Q96EB6 | CYP2C9 | P11712 |
| ALOX15 | P16050 | CYP2C19 | P33261 |
| MTOR | P42345 | SLC6A4 | P31645 |
| SIGMAR1 | Q99720 | CACNA2D1 | P54289 |
| AKR1A1 | P14550 |  |  |
