# Supplementary material for: Systematic Elucidation of the Mechanism of Sappan Lignum in the Treatment of Diabetic Peripheral Neuropathy Based on Network Pharmacology
Source: Evid Based Complement Alternat Med. 2021 Jun 29;2021:5528018. doi: 10.1155/2021/5528018 (PMC8263209; doi:10.1155/2021/5528018)
Supplement: Supplementary Materials — Table S1: the information of 51 intersection targets. Table S2: molecular docking scores (kcal/mol). [file 5528018.f1.zip › 5528018.f1/Table S2.docx]

**Table S2 Molecular docking scores (-kcal/mol)**

| **PDB(ID)** | **SM32** | **SM18** | **SM20** | **SM27** | **Gene name** |
| --- | --- | --- | --- | --- | --- |
| 3o96 | -7.654 | -7.765 | -7.905 | -7.992 | AKT1 |
| 4qtb | -7.526 | -6.976 | -6.927 | -7.772 | MAPK3 |
| 3il8 | -7.545 | -7.137 | -7.843 | -7.412 | CXCL8 |
| 7kpb | -8.262 | -9.587 | -7.735 | -8.053 | TNF |
| 5c1m | -7.375 | -7.095 | -7.092 | -8.029 | OPRM1 |
| 5wbh | -7.125 | -6.942 | -6.947 | -8.023 | MTOR |
| 4zia | -9.035 | -7.029 | -7.067 | -8.982 | STAT3 |
| 4yr8 | -8.332 | -7.249 | -7.149 | -7.494 | MAPK8 |
| 4ig9 | -7.242 | -6.824 | -6.853 | -8.016 | SIRT1 |
| 3q6n | -7.114 | -6.953 | -7.101 | -6.343 | HSP90AA1 |
